# tDCS Facilitation of Picture Naming: Item-Specific, Task General, or Neither?

**DOI:** 10.3389/fnins.2018.00549

**Published:** 2018-08-10

**Authors:** Joshua S. Payne, Marie-Josèphe Tainturier

**Affiliations:** ^1^Bilingual Aphasia Lab, School of Psychology, Bangor University, Bangor, United Kingdom; ^2^Centre for Research on Bilingualism, Bangor University, Bangor, United Kingdom

**Keywords:** transcranial direct current stimulation, lexical retrieval, picture naming, repetition priming, generalization, inferior frontal gyrus, superior temporal gyrus

## Abstract

The aim of the present study was to clarify the conditions under which anodal tDCS applied to left hemisphere language sites may facilitate picture naming latencies in healthy young adults. We built upon previous studies by directly testing for item-specific and generalized effects of tDCS through manipulation of item-familiarization and through testing for both online and offline effects of stimulation, in the same paradigm. In addition, we tested for the robustness of these effects by comparing two left hemisphere sites critical for lexical retrieval. Twenty-eight healthy young adults completed two testing sessions receiving either anodal (1.5 mA, 20 min) or sham stimulation (1.5 mA, 30 s) in each session. Half of the participants received tDCS over the left inferior frontal region and the other half over the left posterior superior temporal region. All participants were asked to a name a set of pictures and their response latencies were compared at three time points (before, during, and after the end of stimulation). The stimulus set was constructed so that some items were presented at all time points, some before and after stimulation, and some during stimulation only. A parsimonious linear mixed effects model (LMM) revealed robust repetition priming effects as latencies were reliably faster for previously named items in all conditions. However, active tDCS did not produce any additional facilitation in relation to sham, and even led to slower performance in the IFG group when the stimulated items differed from those tested at baseline and post-test. Our findings add to the present debate about the efficacy of single-session tDCS for modulation of lexical retrieval in healthy young adults. We conclude that future research should take a more systematic, step-wise approach to the application of tDCS to the study of language and that more sensitive experimental paradigms, which include a training element, are more adapted to the study of cognitive processes in populations with optimal levels of cortical excitability.

## Introduction

The goal of the present study was to test whether transcranial direct current stimulation (tDCS) effects on confrontation naming in healthy young adults reflect task general or item-specific effects. We aimed to gain a better understanding of how stimulation site and stimulus presentation may interact with expected online and offline effects of tDCS – namely facilitation of RTs – in line with previous reports. Observing either item-specific or task-general effects would shed light onto the nature of state-dependent effects of tDCS and provide potential insight for application to treatment paradigms.

Transcranial direct current stimulation is a safe non-invasive brain stimulation technique that has grown increasingly popular as a cognitive neuroscience technique over the past two decades (Dubljević et al., 2014). Compared to other neuroscience techniques (e.g., TMS, fMRI), tDCS is comparatively inexpensive, it is portable, and the proposed mechanisms of action are potentially applicable to multiple domains of cognition and neurorehabilitative treatments (e.g., Cappon et al., 2016). In commonly used setups, weak, constant currents (1–2 mA) are passed into the brain via two (or more) electrodes applied to the scalp for up to 30 min (for technical overview: Woods et al., 2016). Neurons in the path of current flow are thought to be affected in a somewhat polarity specific manner. Anodal (positive) stimulation results in relative depolarization, increasing the likelihood that the neurons will fire, whereas cathodal (negative) stimulation, would hyperpolarize the resting threshold, decreasing firing rates. Applied to task-critical regions, tDCS is thought to increase or decrease the likelihood of long term potentiation/depression, offering the potential to directly manipulate Hebbian plasticity, critical for skill enhancement and rehabilitation (Murphy and Corbett, 2009).

There is increasing evidence that tDCS can be an effective adjunct to behavioral methods in the cognitive rehabilitation of neuropsychological conditions such as aphasia (e.g., Vallar and Bolognini, 2011). However, the efficacy of tDCS for manipulation of cognition in healthy adults is less clear (Horvath et al., 2015; Price et al., 2015; Westwood and Romani, 2017; Klaus and Schutter, 2018). Considerable methodological variability between studies of similar cognitive processes and limited investigative work regarding critical parameters within specific cognitive domains, has contributed to the growing perception that tDCS may be unreliable. Protocols applied to the study of higher-order cognition are extrapolated from studies of the motor system that may not translate in a straightforward manner (Jacobson et al., 2012; Berryhill et al., 2014). The myriad factors that are likely to affect behavioral outcomes in tDCS studies (e.g., timing, stimulation sites, and individual variability) are still poorly understood (for discussion Krause and Cohen Kadosh, 2014).

One area that has been subject to considerable debate is the study of language processing in healthy adults. In a broad meta-analysis of single session tDCS studies of cognition, Horvath et al. (2015) concluded there was no reliable effect of tDCS in any domain, including language. However, their methodology was criticized (Antal et al., 2015; Price and Hamilton, 2015; cf. Horvath, 2015). A re-analysis by Price et al. (2015) demonstrated significant positive effects of tDCS immediately following the stimulation period. One difficulty in interpreting these data is that there is considerable variability between studies, not only in terms of tDCS methodology, but also in terms of the specific language processes being investigated.

The present study focused on the effect of tDCS on word retrieval during confrontation naming in healthy, young adults. Picture naming tasks are commonly administered to probe lexical retrieval in healthy adults and as a focus for remediation of anomia in aphasia. Although picture naming is a simple task, it does involve a complex set of processes. Following visual analysis, the semantic features of a concept are activated from the picture, and in turn activate the lexical representation of the corresponding word form, before activation of phonetic features and motor programs for production (e.g., Rapp and Goldrick, 2006). The accuracy and speed of confrontation naming is highly variable across individuals, and is influenced by factors such as age, level of education and age of acquisition of the test language (e.g., Tombaugh and Hubiey, 1997). Naming performance also varies across items as a function of a number of factors such as lexical frequency, name agreement and semantic density (e.g., Alario et al., 2004; Rabovsky et al., 2016). In addition, it is widely accepted that activation of a set of semantic features leads to the activation of not only the best match lexical representation but also to a larger set of semantically related words, resulting in a noisy selection process (e.g., Oppenheim et al., 2010). In principle, applying anodal tDCS to cortical sites critical for lexical retrieval (e.g., left IFG, left STG; Price, 2012) during a skilled task like naming will result in suppression of noise and maximize the signal for selection of the correct object naming, leading to facilitation of naming responses (Miniussi et al., 2013, p. 1707).

Although much of the literature regarding tDCS and word retrieval has focused on semantic interference paradigms (for critical review, Meinzer et al., 2016), several studies on confrontation naming in healthy adults have reported faster responses following anodal tDCS applied to left-hemisphere sites (cf. Rosso et al., 2014 for cathodal over right IFG). Sparing et al. (2008) published the first tDCS study of picture naming. They observed facilitation immediately following the cessation of online anodal stimulation applied to left posterior temporal regions (e.g., Wernicke’s area), However, RTs returned to baseline by 5 min post-tDCS. In another study, Fertonani et al. (2010) explored the effect of offline stimulation (stimulation without a concurrent task) applied to left dorsolateral pre-frontal cortex (DLPFC, 2 mA) before the administration of an object and action naming task. RTs were faster immediately following anodal stimulation, as compared to cathodal and sham but facilitation was not specific to grammatical class. In a follow-up study utilizing the same object-action naming task and stimulation site, Fertonani et al. (2014) compared the effect of online and offline stimulation, in younger and older adults. In older adults, facilitation was observed in the online protocol only. In younger adults, both online and offline stimulation led to faster responses. However, in Wirth et al. (2011) picture naming facilitation in young adults was observed during online stimulation of the DLPFC but not offline. Taken together these findings support potential reproducibility of tDCS effects on picture naming (see also Klaus and Schutter, 2018) although these effects might be short-lived and limited to on-line protocols. Online stimulation may promote plasticity for task-specific processes, enhancing LTP/LTD in a direct way – so-called state-dependency (Silvanto et al., 2008; Miniussi et al., 2013), a rationale that is prevalent in anomia treatment studies (for review, Crinion, 2016). In contrast, offline protocols may have a broader effect on cortical “readiness” prior to task performance, resulting in general up-regulation of processing.

Despite these positive reports, a recent meta-analysis of word retrieval studies in healthy participants concluded that the effects of tDCS are not statistically reliable (Westwood and Romani, 2017) whatever the stimulation site or protocol. It also highlighted that a publication bias for positive findings may have led to an over-estimation of the influence of tDCS on cognitive processing. Consistent with this view, the same research group failed to observe any effects of tDCS across four word retrieval experiments (Westwood et al., 2017; but see Gauvin et al., 2017).

Nevertheless, it is probably too soon to abandon the paradigm altogether. As we have seen, it is difficult to compare studies directly as they vary on multiple dimensions, some of which may be determinant. In addition to the stimulation parameters themselves (e.g., intensity, duration, electrode placement), there are variations in important aspects of the design of different studies that have not been systematically evaluated. Amongst these, some key factors are: (1) the stimulation sites, (2) whether the stimulation is delivered on-line vs. off-line (i.e., during vs. before the execution the task assessed), (3) when potential changes in performance are assessed (i.e., in relation to a baseline or not, during vs. post-stimulation, how long after stimulation), (4) whether the study measures changes within-participants or across groups (active vs. sham). In addition, an important factor concerns the specificity of the expected effects of tDCS stimulation: should facilitation be restricted to specific trained words or would one expect to observe a more global improvement in lexical retrieval performance, generalizing across items and possibly tasks? Prior studies do not inform this issue as they either presented the same items in each of the conditions (Holland et al., 2011) or else provided over-training before naming; Sparing et al. (2008) with the aim of reducing variance prior to stimulation. In the absence of a condition specifically designed to assess generalization (cf. Fertonani et al., 2010, 2014, for a general effect of tDCS to action and object naming), positive results (when they occur) are equally compatible with the hypothesis of item-specific effects or of general up-regulation of the lexical retrieval process (e.g., Holland et al., 2011). A related question is whether the activation of specific items prior to stimulation is a necessary condition to observe tDCS effects. Resolving these questions may shed light on the mechanisms of action of tDCS in various contexts. It is likely that tDCS effects will interact with the network state at the point of stimulation, which could alter performance during and/or for some time after stimulation.

The goal of our study was to gain a better understanding of the effects of tDCS on word retrieval in healthy, young adults. Participants were asked to name sets of pictures and their naming latencies at “baseline” (pre-stimulation) were compared to their latencies during stimulation as well as 15 min post-stimulation. The study was designed to: (1) examine if potential effects would be item-specific vs. task general and (2) to directly compare the effects of tDCS at two cortical sites.

To address the question of item-specificity, we created three stimulus lists that were presented to all participants at different time points. List A items were named at all timepoints (before, during, and after the stimulation period). List B items were named before and after stimulation but not during. Finally, List C items were named during stimulation only. This allows us to examine if potential effects of tDCS *during* stimulation would be specific to items pre-activated at baseline and if effects *post-*stimulation would be specific to items produced during the prior stimulation period. On the other hand, if tDCS leads to a general up-regulation of lexical retrieval then we should see a comparable reduction of naming latencies across lists in the active condition relative to sham.

Our second goal was to directly contrast the effects of tDCS at two sites, the left IFG and left pSTG, chosen for their well-established role in lexical retrieval (e.g., Price, 2012) and salience as tDCS target sites in the treatment of stroke-induced aphasia (e.g., Crinion, 2016); In addition, positive tDCS effects have been reported at both sites in healthy young adults but have not been directly compared. In the present study we conducted the same experiment in two groups of participants, who received anodal and sham stimulation to either the left IFG or left pSTG. Comparable findings at both sites would lend support to the idea that tDCS can facilitate picture naming at multiple sites within the lexical retrieval network. On the other hand, the STG and IFG are thought to play different roles within this network, one common hypothesis being that temporal regions are involved in lexical storage while frontal sites would be more involved in the control of the relative activation of competing lexical candidates (Schnur et al., 2009; Riès et al., 2015; Piai and Knight, 2018).

## Materials and Methods

### Participants

Twenty-eight monolingual English speakers (Male = 13; *M*_AGE_ = 24.22, *SD* = 3.45), recruited from the student population at Bangor University took part in this study. Half of the participants were randomly assigned to the IFG condition and half to the pSTG condition. There were no significant differences between groups in terms of age or years of education (*p* > 0.14). All participants were right-handed, with no history of dyslexia or brain injury, and reported no contraindications for tDCS, as assessed by an in-house screening questionnaire. All data were collected between August 2014 and March 2015.

### Ethical Considerations

All stimulation and experimental protocols were reviewed by the Bangor Brain Stimulation Committee, prior to approval from the School of Psychology’s REC (2014-12525-A11682). All subjects gave written informed consent in accordance with the Declaration of Helsinki.

### Stimuli

Stimuli were black and white line drawings (300 px × 300 px) from a 416-item subset of the English (US) version of the International Picture Naming Project (IPNP); Szekely et al. (2004) and Holland et al. (2011) have previously used subsets from this database in tDCS studies exploring picture naming in Italian and English, respectively.

We included items from the IPNP that had a lenient name agreement of 100%, i.e., accepting dominant names, synonyms, and morphophonological variants of the target. For example, both bike and bicycle are acceptable answers. Items with complex morphological structure were excluded (i.e., ice cream cone). We divided this subset into three 43-item lists matched on CELEX frequency (Baayen et al., 1995) and on the Zipf frequency (1–7 scale) and contextual diversity (CD) values from the SUBTLEX-UK corpus (van Heuven et al., 2014). Items with a higher lexical frequency or with a greater contextual diversity are named more quickly (Adelman et al., 2006). On average, object names were of a moderate frequency (see **Table 1**), and they ranged from low to high based on both frequency counts [CELEX (natural logarithm) = min: 0.00; Max 6.08; SUBTLEX (Zipf) = Min = 3.09; Max: 5.44]. mA multivariate analysis of variance (MANOVA) with List (A, B, C) as an independent factor conducted on CELEX frequency, Zipf frequency and on the log transformation of CD was non-significant, Wilks’ λ = 0.99, *F*(6, 250) = 0.27, *p* = 0.95, suggesting that the lists were well matched across all three variables. In addition, items from each of the three CDI (Communicative Developmental Index; Fenson et al., 1994) age of acquisition categories, included in the IPNP, were distributed evenly across lists, χ^2^(4, *N* = 129) = 0.07, *p* = 0.99. Kruskal–Wallis *H*-tests showed no significant difference in mean ranks for syllables [*H*(2) = 0.43, *p* = 0.81], letter length [*H*(2) = 0.25, *p* = 0.88] or phoneme length [*H*(2) = 0.87, *p* = 0.65]. Finally, the three lists were matched on initial phoneme category (e.g., stop consonants, fricatives) to minimize effects of articulatory planning time (Rastle and Davis, 2002). All matching statistics are presented in **Table 1**. A pilot study with 10 young adults confirmed that the three sub-lists were well matched for difficulty as naming latencies were equivalent across lists.

**Table 1:** Matching statistics for variables in each target list (Means and SD).

	*n*	Complex initial phoneme (*n*)	Ln celex freq.^a^	Zipf freq.^b^	CD^a^	Syllables^c^	Letters^c^	Phonemes^c^	RT	CDI AoA categories% (*n*)	
**A**	43	11	3.37	4.41	0.093	1.33	4.72	3.84	713.5	8–16 months	46.5% (20)
			(1.40)	(0.48)	(0.105)	(0.52)	(1.16)	(1.13)	(106.66)	17–30 months	14% (6)
										>30 months	39.5% (17)
**B**	43	11	3.03	4.32	0.089	1.42	4.79	3.95	703.06	8–16 months	46.5%(20)
			(1.36)	(0.60)	(0.105)	(0.66)	(1.39)	(1.29)	(102.05)	17–30 months	14% (6)
										>30 months	39.5% (17)
**C**	43	11	3.20	4.36	0.076	1.44	5.00	4.16	718.5	8–16 months	44.2% (19)
			(1.05)	(0.60)	(0.074)	(0.67)	(1.62)	(1.46)	(66.14)	17–30 months	14%(6)
										>30 months	41.9% (18)

*Freq., Frequency; CD, Contextual Diversity; CDI, MacArthur Communicative Index. ^*a*^Natural logarithm applied. ^*b*^Calculated using theZipf formula provided for SUBTLEX-UK (van Heuven et al., 2014). ^*c*^Kruskal–Wallis H calculated using mean ranks.*

### Picture Naming Task

Participants completed three picture naming tasks – Pre, During, and Post stimulation – in each of two sessions. Following a 10-item practice (non-overlapping with stimulus lists), participants named pictures from two of the three 43-item lists (Pre: AB; During: AC; Post: AB) for each naming task. A trial was made up of a 1000 ms fixation cross followed by a picture of a single object, which remained on screen until the participant’s response triggered the voice key. The experimenter coded response accuracy on a serial response box, which triggered the next trial. The whole session was recorded. A 300 ms beep sounded with the onset of the picture to enable manual reference of response times at a later stage.

List A was presented at all three time points and the items in this set were subject to repetition plus online stimulation. List B was presented pre- and post-stimulation only, and List C during stimulation only. List B is subject to repetition priming post-stimulation but not to direct stimulation. Items in List C were novel in relation to items in List A or B, and not subject to repetition priming within a session. The BCB set allowed us to test for offline tDCS effects on repeated B items and List C provided a direct test of item-specific versus generalized effects of online tDCS. The items from both lists presented at any time point (Pre: AB, During: AC, and Post: AB) were intermixed and presented in a unique random order for every instance of the task. All participant response times were manually recoded using Praat (Boersma, 2006).

### Transcranial Direct Current Stimulation

**FIGURE 1:**
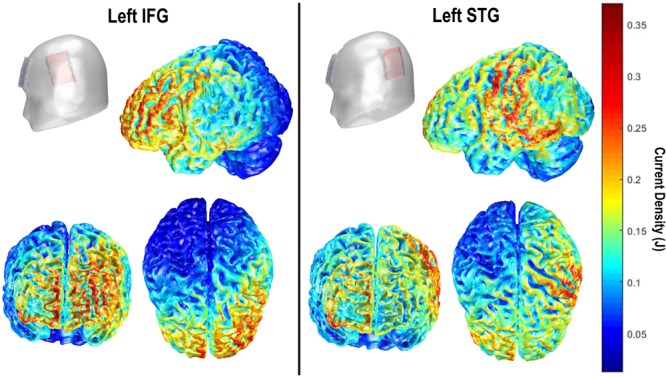
COMETS2 (Lee et al., 2017) current density (J) models for left IFG **(Left)** and left STG montages **(Right)**.

Transcranial direct current stimulation (tDCS) was delivered single-blind, using a battery powered, constant-current stimulator (NeuroConn DC-Stimulator Plus, Rogue Resolutions). Current was delivered through a pair of 35 cm^2^ conductive rubber electrodes, inserted into sponges moistened with 0.75% saline solution to improve conductivity and promote participant comfort (Dundas et al., 2007). Each participant received two sessions of tDCS (active or sham), separated by at least a week. For the IFG group, the anode was centered over FC5 which overlies the inferior frontal gyrus (e.g., Jurcak et al., 2007; Koessler et al., 2009; Holland et al., 2011). For the STG group, the anode was centered over CP5 overlying the LSTG in accordance with a previous study (c.f., Sparing et al., 2008). In both groups the reference electrode was placed over the right supraorbital area. In active sessions, current was delivered at 1.5 mA (Current Density = 0.043 mA/cm^2^) for a total of 20 min. Stimulation duration was informed by a general overview of the tDCS literature, which suggested a somewhat standard application of 20 min online stimulation. We chose 1.5 mA as a trade-off between likelihood of observing a tDCS effect and participant comfort/blinding, in response to reports of ineffective blinding at higher intensities (O’Connell et al., 2012; Davis et al., 2013). In sham sessions, stimulation was ramped up, left on for 30 s and ramped down. We employed a ramp-on/off of 15 s, in both stimulation sessions, in accordance with previous studies (e.g., Holland et al., 2011). Impedance was below 5 kΩ before commencing stimulation in all participants. **Figure 1** shows models of current density for both electrode montages created using the COMETS2 tool for MATLAB (Lee et al., 2017).

### Procedure

**FIGURE 2:**
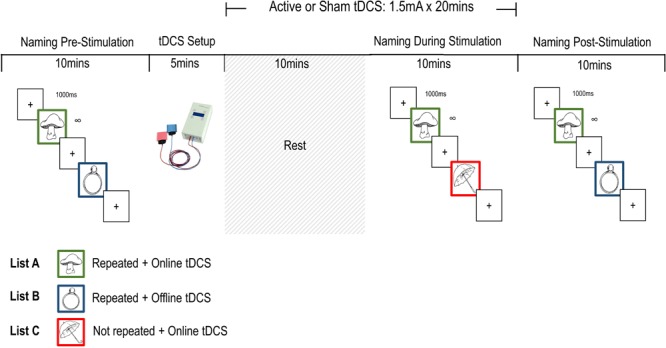
Single-session procedure outline.

Participants took part in two 1 h sessions, separated by at least 7 days (*M* = 9.90, *SD* = 3.67). Sham or anodal stimulation was delivered in each session and the order of stimulation was counterbalanced across participants. At the beginning of the first session participants were given a brief overview of tDCS procedures, and an opportunity to ask questions, before completing the screening questionnaire and giving informed consent. The stimulator was switched on and the electrodes were placed on the scalp before the first naming task to encourage habituation, reduce impedance, and prevent distraction between tasks. Participants named 86 pictures before stimulation (AB), after 10 min of stimulation (AC), and around 5 min after the end of stimulation (AB). Approximately 1 min into stimulation the sensations questionnaire was administered. At the end of the second session, we asked participants to indicate whether they thought they had received active or sham stimulation in either session before debriefing them. An overview of the procedure is presented in **Figure 2**.

### Design

We employed a mixed design with Site (IFG, STG) as a between-subject factor and Stimulation (Sham, Active), time point (Pre, During, and Post), and List (AAA, BCB) as within-subject factors. The dependent variable in this study was onset naming latencies.

### Analysis

All analyses were conducted in Microsoft Open R 3.3.2 (Microsoft R Application Network, 2014), a distribution of the R software (R Core Team, 2016), optimized for multi-core processing.

#### Blinding and Sensations

We assessed blinding at the end of the study, by asking participants whether they thought they had received active or sham stimulation in either session, asking them to guess if unsure. Blinding was not intact as correct discrimination was significantly above chance (0.5) for both groups [IFG: 78.57% (11/14), χ^2^(1) = 4.57, *p* = 0.061; STG: 85.71% (12/14), χ^2^(1) = 7.14, *p* = 0.011]. Overall, 82.14% (23/28) of participants could discriminate accurately between Active and Sham sessions, χ^2^(1) = 11.57, *p* = 0.001.

In addition, we measured the presence and intensity of after effects during stimulation in each session, using a nine-item questionnaire, modeled on Poreisz et al. (2007) and Brunoni et al. (2011). Participants indicated the incidence of a sensation by choosing yes/no and then rated the intensity of sensations on a five-point scale (1 = Very Mild, 5 = Very Strong).

All sensations were rated within the mild to moderate range. Supplementary Table S1 provides intensity and incidence ratings for all sensations with accompanying radar plots for visual comparison across sites. The intensity of Tingling, Heating and Pain was greater during active stimulation compared to sham in the IFG group (all *p* ≤ 0.048), with a marginal effect on Itching (*p* = 0.074). None of the comparisons between sham and active conditions for the STG group were significant. Comparison of overall mean discomfort ratings between groups was non-significant. Anecdotally, it is likely that differences in perceived *duration* of sensations was responsible for suboptimal blinding in the present study, although the Poreisz et al. (2007) questionnaire does not ask about such information.

#### Picture Naming Tasks

Responses were coded as correct if the participant produced the dominant name, a morphophonological variant, or a synonym of the dominant name. Incorrect responses, and microphone errors (e.g., false trigger, failed trigger, external noise overriding the waveform) that could not be reconciled from the recording, were excluded from analyses. Of the 14,448 data points included in our dataset, 1.70% (*n* = 245) of trials were lost to microphone errors, with an additional 0.71% (*n* = 102) excluded as incorrect naming responses. As error rates were less than 1% overall, we limited our analyses to response time data from correct trials only. This left 14,101 (97%) data points for inclusion in the analysis.

#### Parsimonious Linear Mixed Effects Models

Parsimonious linear mixed effects models (LMMs) were conducted on inverse reciprocal transformed RTs (InvRT = -1000/RTs, Baayen et al., 2008; Kliegl et al., 2010) with lme4 1.1–12 (Bates et al., 2014) and RePsychLing 0.0.4 packages (Baayen et al., 2015) as outlined by Bates et al. (2015) models with untransformed RTs produce similar results). Models included centered, categorical fixed effects of Site (IFG, STG), Stimulation (Sham, Active) and List^1^ (AAA, BCB) as well as a centered, treatment-coded, categorical predictor of time point (Pre, During, Post), yielding two contrasts: Pre-During and Pre-Post. The two contrasts allowed us to test for online and offline effects of tDCS, respectively. In the final model, 321 trials (2.27%) were excluded as outliers (absolute standardized residual >2.5), and the model was refit to improve normality of residuals in accordance with a minimal *a priori* data trimming approach (Harald Baayen and Milin, 2010). The random effects in the final parsimonious model included intercepts for both participants and items, within-item slopes of site and time point, and within-participant slopes of time point, Stimulation and their interaction. Random effects correlations were included in the final model as they improved the model fit. Beta weights and standard errors are presented alongside Wald approximated *p*-values.

## Results

**FIGURE 3:**
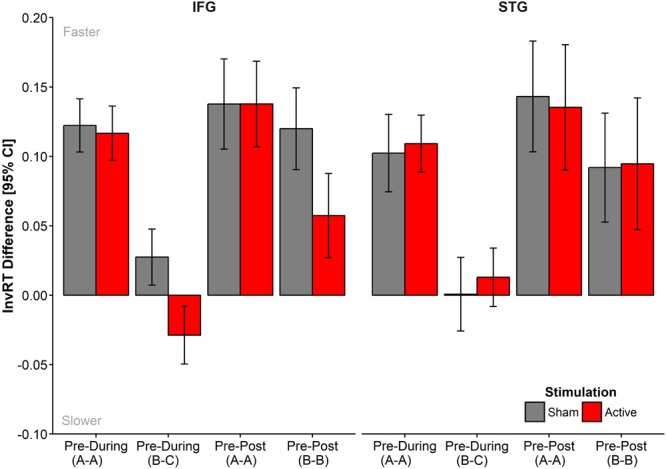
Mean difference in InvRTs for each of the time point contrasts, Pre-During and Pre-Post, entered into the model. Negative values reflect slower response times at the later time point. Data was generated based on fitted data extracted from final model and aggregated across participants. Means, adjusted for intra-subject variability, plus 95% CIs were extracted according to Morey (2008) and implemented with the summarySEwithin() function in R (Chang, n.d.).

**Figure 3** displays the mean differences for the Timepoint contrasts, Pre-During, and Pre-Post, split by List, Site, and Stimulation. The full model summary from the final model is presented in Supplementary Table S2. **Table 2** includes mean untransformed and inverse reciprocal transformed onset naming times (correct trials only) and standard deviations for all conditions, after exclusion of outliers following model criticism.

**Table 2:** Mean (standard deviation) untransformed (RT) and transformed (InvRT) correct onset naming latencies for each of the conditions.

			Sham			Active		
		Pre	During	Post	Pre	During	Post
**RT**		
	IFG	A	661 (135)	612 (111)	610 (127)	658 (137)	612 (114)	606 (117)
		B	669 (168)		617 (129)	652 (158)		631 (140)
		C		654 (136)			669 (156)	
	pSTG	A	717 (184)	661 (163)	641 (151)	699 (153)	648 (152)	637 (148)
		B	725 (231)		664 (172)	711 (194)		658 (162)
		C		715 (212)			706 (208)	
**InvRT**		
	IFG	A	–1.56 (0.25)	–1.68 (0.24)	–1.69 (0.26)	–1.57 (0.26)	–1.68 (0.23)	–1.70 (0.24)
		B	–1.57 (0.28)		–1.68 (0.26)	–1.60 (0.29)		–1.65 (0.26)
		C		–1.58 (0.27)			–1.56 (0.27)	
	pSTG	A	–1.47 (0.28)	–1.58 (0.25)	–1.63 (0.25)	–1.49 (0.26)	–1.61 (0.26)	–1.64 (0.25)
		B	–1.48 (0.31)		–1.58 (0.28)	–1.49 (-1.53)		–1.59 (0.26)
		C		–1.49 (0.28)			–1.51 (0.28)	

*Data were extracted from the final dataset following model criticism to remove influential points.*

The final model revealed no significant main effects of List (AAA, BCB) or Stimulation (Anodal, Sham), but there was a trend for naming latencies to be slower in the STG group [*M* = -1.55 (∼645 ms)^2^, *SE* = 0.05] compared to the IFG [*M* = -1.63 (∼614 ms)^2^, *SE* = 0.04; β = -0.10 ± 0.02, *t* = 1.76, *p* = 0.090]. As expected, naming latencies were shorter at later time points compared to Pre, reflecting priming to repeated sets (Pre-During: β = -0.06 ± 0.02, *t* = -3.61, *p* < 0.001; Pre-Post: β = -0.12 ± 0.02, *t* = -6.65, *p* < 0.001). Also as expected, there was an interaction between time point and list in the Pre- vs. During comparisons (Pre-Dur^∗^List: β = 0.11 ± 0.03, *t* = 3.62, *p* < 0.001), as only the repeated set (List A) was subject to priming. In contrast, the naming latencies for set C (presented during stimulation only) were comparable to the latencies obtained for set B pre-stimulation. In the pre-post contrasts, we observed priming for both lists, but the reduction in naming latencies was larger for set A than for Set B (Pre-Post^∗^List: β = 0.05 ± 0.02, *t* = 2.95, *p* = 0.004) reflecting the fact that at post-test List A was being presented for the third time while List B was only presented for the second time.

Crucially, there was no evidence that active stimulation facilitated naming latencies in relation to sham stimulation and this irrespective of stimulation site, as indicated by non-significant higher-order interactions. As can be seen in **Figure 3**, naming latencies were *slower* in the active condition in the IFG group for both List C (pre-during contrast) and List B (pre-post contrast). No such effects were visible in the STG group nor for List A. There was a marginally significant four-way interaction including the Pre-Post contrast (Site^∗^Stimulation^∗^Pre-Post^∗^List: β = -0.06 ± 0.04, *t* = -1.75, *p* = 0.081) but not the Pre-During contrast, (Site^∗^Stimulation^∗^Pre-During^∗^List: β = -0.05 ±0.04, *t* = -1.36, *p* = 0.17).

To follow up on the trend in the interaction, we conducted simple effects analyses by fitting a separate LMM for the IFG group, for List B and C items only (see Supplementary Table S3), which showed a disruptive influence of active tDCS on both List C (During) and List B (Post) items (Stimulation^∗^Pre-During_IFG_: β = 0.05 ± 0.02, *t* = 2.58, *p* = 0.011; Stimulation^∗^Pre-Post_IFG_: β = 0.06 ± 0.03, *t* = 2.25, *p* = 0.038).

We carried out additional analyses in order to rule out that our findings may have stemmed from the action of confounded variables. The overall pattern of results remained unchanged when we included either blinding (Intact, Not-intact) or stimulation order (Sham first or second) as categorical covariates into the final model, as well as when including word frequency or participant baseline naming latencies as continuous covariates (see Supplementary Table S2).

## Discussion

The aim of the present study was to clarify the conditions under which anodal tDCS applied to left hemisphere language sites may facilitate picture naming latencies in healthy adults. We built upon previous studies by directly testing for item-specific and generalized effects of tDCS through manipulation of item-familiarization and through testing for both online and offline effects of stimulation, in the same paradigm. In addition, we tested for the robustness of these effects by comparing two left hemisphere sites critical for lexical retrieval, the left IFG and pSTG. We also improved on earlier studies with careful matching of key stimulus and participant characteristics, coupled with analyses that take variability across both participants and stimuli into account.

In summary, our results provide a robust replication of the classical repetition priming effect as naming latencies were reliably faster for previously named sets in all conditions. However, active tDCS did not produce any additional facilitation. Given these null findings, whether facilitation is best characterized as an item-specific or a generalized effect becomes a moot point. We did observe an effect of active stimulation but it was a negative one: In the IFG group, naming latencies were slower at post-test for set B items – those that were presented at pre- and post-test, with the C set intervening during stimulation. This finding was not predicted and should not be given too much weight. Direct investigation of this effect or replication of the present study should be conducted. Tentatively, our results may suggest that tDCS could interfere with retrieval when the lexical system has just been primed with a different word list. It is interesting that this apparent interference effect of active tDCS is limited to stimulation of the LIFG which has been linked to the modulation of patterns of relative activation amongst competing lexical units (e.g., Moss et al., 2005; Hofmann and Jacobs, 2014; Meinzer et al., 2016). There are several possible reasons why we did not observe a facilitatory effect of tDCS in our study. First, it could be argued that we might have observed tDCS facilitation if we had used higher amplitude stimulation. We chose to deliver 1.5 mA tDCS as it would appear to be the highest intensity at which blinding can be preserved (Ambrus et al., 2010, 2012; Kessler et al., 2012; O’Connell et al., 2012; Davis et al., 2013; Wallace et al., 2016). In their meta-analysis, Westwood and Romani (2017) reported that current intensity, that varied between 1 and 2 mA in the studies included, did not predict facilitation effects. Thus, it seems unlikely that our results are due to us using too weak an intensity of stimulation. Further step-wise evaluation of key methodological parameters within specific cognitive domains, such as titration of stimulation amplitude would be a useful development, as protocols from the motor domain do not necessarily translate to other cognitive domains (Jacobson et al., 2012; Berryhill et al., 2014).

A potential problem is that even at a relatively low intensity (1.5 mA) we could not achieve full blinding, with 85% of participants correctly discriminated between the two conditions when asked to guess at the end of the second session. This was surprising given that difference in stimulation intensity between sham and anodal conditions in this study was comparable to previous studies who claimed to have achieved successful blinding. This suggests that if using intensities greater than or equal to 1.5 mA a between-subject design would be a more suitable option, as cross-over designs are likely to make differences in sensations more noticeable. On the other hand, a downside of between-subject designs is that differences between active and sham may be due to an insufficient control of participant characteristics across groups. Be this as it may, it is far from clear how participants’ relative awareness of stimulation conditions may have affected performance in our study. One possibility is that it could have increased distractibility during stimulation. However, if this were the case one would expect to observe an increase in naming latencies during and possibly after active tDCS irrespective of stimulation location, in contrast to what we observed.

Thus, taking our results at face value, our study joins an increasing number of recent reports and meta-analyses that highlight the considerable heterogeneity of non-invasive brain stimulation effects on word production in healthy young adults (Westwood and Romani, 2017; Klaus and Schutter, 2018) One likely explanation is that, generally speaking, tDCS may not be effective when performance is already close to being optimal, as in healthy young adults performing relatively easy tasks. In the present study (as in others), the stimuli were all familiar enough to be named with high accuracy in order to optimize the analyses of naming latencies. This may have left insufficient room for additional tDCS-driven facilitation in our groups of highly educated healthy young adults. From a stochastic resonance perspective, this would correspond to a low-noise, high-signal condition, leading to limited efficacy of tDCS (Fertonani and Miniussi, 2017). This ratio would have been reduced even further due to the repeated presentation of stimulus sets leading to strong priming effects. In such conditions, any effects would be small at best, and a much larger number of participants and items than typically used would be needed to reach sufficient power. This being said, a lack of power is unlikely to account for our results since there were no numerical trend for tDCS facilitation is any of our conditions. Consistent with this interpretation, tDCS effects in similar paradigms are much clearer in populations with sub-optimal levels of cortical excitability, such as in aphasia following brain damage (for reviews see Cappon et al., 2016; Crinion, 2016; Sandars et al., 2016) or in older participants affected by age-related decreases in neural plasticity (e.g., Holland et al., 2011; Ross et al., 2011; Fertonani et al., 2014). Although we did not observe on-line facilitation for unprimed items (List C), our design did not allow us to examine if active stimulation of an unprimed list may lead to off-line improvement at post-test. This would have required presenting List C at post-test, as well as a new list to test for generalization effects if tDCS enhances lexical retrieval more generally. Finally, electrode placement is a central factor that deserves further investigation as differences in electrode placement, however small, may have a significant impact on observed effects (Penolazzi et al., 2013). Our electrode montage was chosen to maximally target the lexical processing network, but alternative montages may prove more effective. Furthermore, stimulation targeting other brain areas may well turn out to be a requirement for observing facilitation effects in young healthy adults. For example, stimulation applied to DLPFC has produced some positive effects (Fertonani et al., 2010, 2014), although this probably reflects an improvement of broader task regulation processes rather than of lexical retrieval *per se*.

However, that is not to say that tDCS should be dismissed as a neuromodulatory device for the study of language processing in healthy adults. Tasks that avoid ceiling effects are perhaps better suited to probing questions about the healthy, young brain. For example, it has been argued that tDCS effects could be limited to studies using training paradigms (Mancuso et al., 2016). Along this line, substantial improvements during anodal tDCS in performance in healthy young adults have been reported in studies of vocabulary learning (Meinzer et al., 2007; Flöel et al., 2008; De Vries et al., 2010; Liuzzi et al., 2010; Fiori et al., 2011; Savill et al., 2015). Similarly, we recently observed improved performance in a foreign language vocabulary learning task with 1mA anodal tDCS applied over the left pSTG. Interestingly, this effect was only observed for participants with relatively lower (though normal) phonological memory abilities while active stimulation showed a tendency to impair performance in translation for participants with higher phonological memory abilities on the easier learning sets (Payne et al., 2017). In addition to their greater sensitivity, a major benefit of learning paradigms is that they are closer to those used with impaired populations, which may lead to further developments relevant to neurorehabilitation. Finally, investigations with potentially more effective electrical waveforms, such as transcranial random noise stimulation (tRNS) or transcranial alternating current stimulation (tACS) may prove fruitful for the modulation of higher order cognition in younger adults (e.g., Paulus, 2011; Snowball et al., 2013; Romanska et al., 2015; Penton et al., 2017).

## Conclusion

In conclusion, this study joins an increasing number of publications in casting doubts about the effectiveness of single session tDCS for improving word retrieval processes in healthy young adults. It remains possible that more reliable tDCS effects may emerge in picture naming and related tasks by administering multiple stimulation sessions, by tweaking stimulation parameters or by increasing sample size. However, this would also considerably increase the ratio of costs to potential benefits, suggesting that moving to more sensitive experimental paradigms, which include a training element could prove more promising.

## Author Contributions

JP has made a dominant contribution to all aspects of the work; this study is part of his doctoral dissertation. M-JT has significantly contributed to all stages of the work presented (from initial design to write-up) except for data collection.

## Conflict of Interest Statement

The authors declare that the research was conducted in the absence of any commercial or financial relationships that could be construed as a potential conflict of interest.

## Footnotes

1 List was coded as a two-level variable (AAA, BCB) to facilitate easy application of contrasts between the two lists presented at each time point.

2 Raw RT estimated on the basis of back-transformation of inverse reciprocal RTs (-1000/InvRT).
