# Supplementary material for: tDCS Facilitation of Picture Naming: Item-Specific, Task General, or Neither?
Source: Front Neurosci. 2018 Aug 10;12:549. doi: 10.3389/fnins.2018.00549 (PMC6095956; doi:10.3389/fnins.2018.00549)
Supplement: Supplementary file 1 [file Table_1.DOCX]

Table S1

**
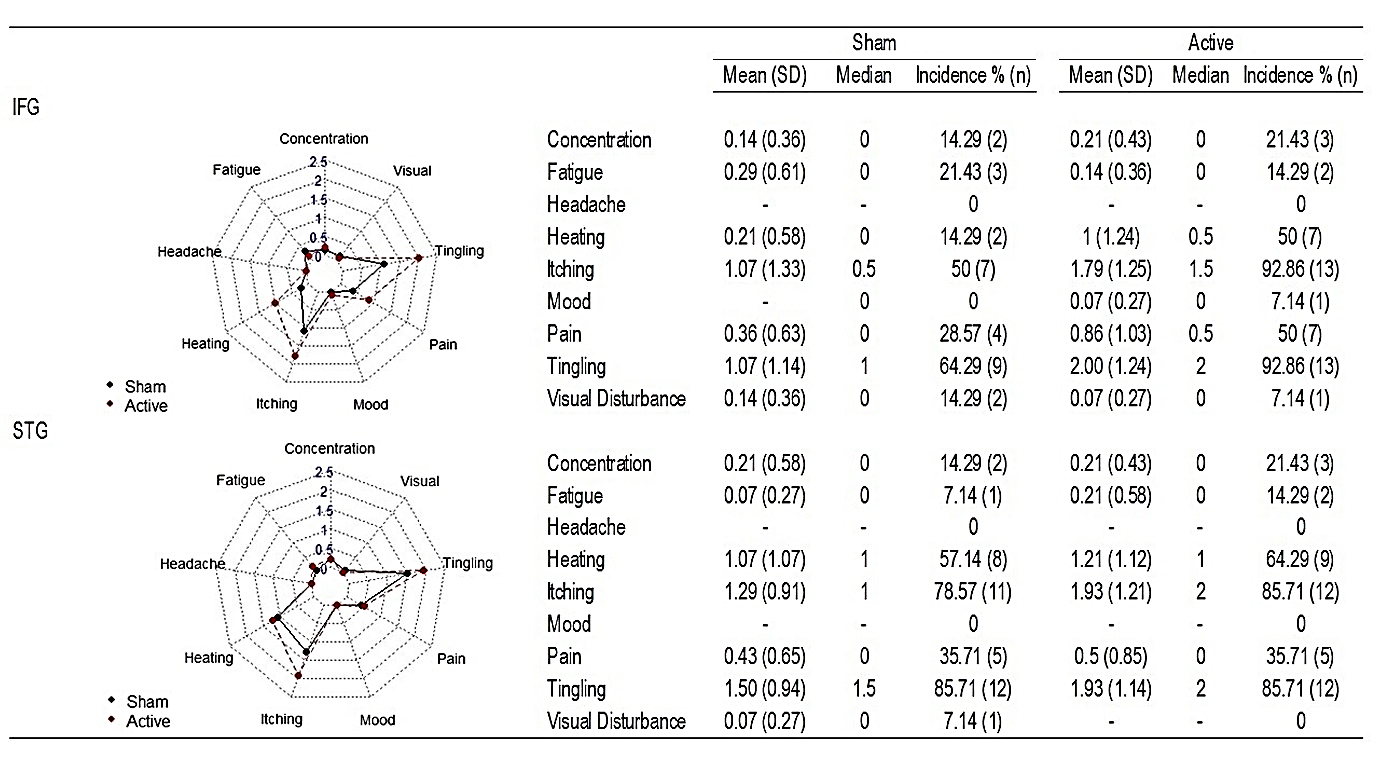
***Mean (SD), Median Intensity and Incidence of Sensations at Onset of Stimulation. Radar plots are included for visual comparison.*

Note: Radar plots created in R using the fmsb package (Nakazawa, 2015).
