# Supplementary material for: tDCS Facilitation of Picture Naming: Item-Specific, Task General, or Neither?
Source: Front Neurosci. 2018 Aug 10;12:549. doi: 10.3389/fnins.2018.00549 (PMC6095956; doi:10.3389/fnins.2018.00549)
Supplement: Supplementary file 2 [file Table_2.DOCX]

Table S2. *Model Summaries for Parsimonious LMM and Supplementary Models with Covariates*

|  |  | Parsimonious LMM | | |  | Parsimonious + Blinding | | |  | Parsimonious + Order | | |  | Parsimonious + Zipf | | |  | Parsimonious + Baseline RT | | |
| --- | --- | --- | --- | --- | --- | --- | --- | --- | --- | --- | --- | --- | --- | --- | --- | --- | --- | --- | --- | --- |
|  |  | B | 95% CI | p |  | B | 95% CI | p |  | B | 95% CI | p |  | B | 95% CI | p |  | B | 95% CI | p |
| Blinding (Not intact, Intact) | |  |  |  |  | 0.019 | -0.12, 0.15 | .785 |  |  |  |  |  |  |  |  |  |  |  |  |
| Session Order | |  |  |  |  |  |  |  |  | 0.127 | 0.03, 0.23 | **.012** |  |  |  |  |  |  |  |  |
| Zipf Frequency | |  |  |  |  |  |  |  |  |  |  |  |  | -0.041 | -0.07, -0.01 | **.017** |  |  |  |  |
| z(Baseline Naming) | |  |  |  |  |  |  |  |  |  |  |  |  |  |  |  |  | 0.155 | 0.15, 0.16 | **<.001** |
| (Intercept) | | -1.526 | -1.59, -1.46 | **<.001** |  | -1.541 | -1.67, -1.41 | **<.001** |  | -1.526 | -1.59, -1.46 | **<.001** |  | -1.526 | -1.59, -1.47 | **<.001** |  | -1.525 | -1.56, -1.49 | **<.001** |
| Site (IFG, STG) | | 0.096 | -0.01, 0.20 | ***.088*** |  | 0.095 | -0.01, 0.20 | ***.093*** |  | 0.096 | -0.01, 0.20 | ***.082*** |  | 0.097 | -0.01, 0.20 | ***.076*** |  | -0.001 | -0.03, 0.03 | .963 |
| Stimulation (Sham, Active) | | -0.018 | -0.08, 0.04 | .547 |  | -0.018 | -0.08, 0.04 | .547 |  | -0.018 | -0.08, 0.04 | .542 |  | -0.018 | -0.08, 0.04 | .543 |  | -0.018 | -0.08, 0.04 | .542 |
| Pre v. During | | -0.06 | -0.09, -0.03 | **<.001** |  | -0.06 | -0.09, -0.03 | **<.001** |  | -0.06 | -0.09, -0.03 | **<.001** |  | -0.06 | -0.09, -0.03 | **<.001** |  | -0.06 | -0.09, -0.03 | **<.001** |
| During v. Post | | -0.116 | -0.15, -0.08 | **<.001** |  | -0.116 | -0.15, -0.08 | **<.001** |  | -0.116 | -0.15, -0.08 | **<.001** |  | -0.116 | -0.15, -0.08 | **<.001** |  | -0.116 | -0.15, -0.08 | **<.001** |
| List (AAA, BCB) | | -0.004 | -0.06, 0.06 | .895 |  | -0.004 | -0.06, 0.06 | .895 |  | -0.004 | -0.06, 0.06 | .895 |  | -0.008 | -0.07, 0.05 | .793 |  | -0.004 | -0.06, 0.06 | .896 |
| Site x Stimulation | | 0.009 | -0.11, 0.12 | .875 |  | 0.009 | -0.11, 0.12 | .875 |  | 0.009 | -0.11, 0.12 | .875 |  | 0.009 | -0.11, 0.12 | .874 |  | 0.009 | -0.11, 0.12 | .874 |
| Site x Pre-Dur | | -0.021 | -0.06, 0.02 | .264 |  | -0.021 | -0.06, 0.02 | .264 |  | -0.021 | -0.06, 0.02 | .258 |  | -0.021 | -0.06, 0.02 | .259 |  | -0.021 | -0.06, 0.02 | .257 |
| Site x Pre-Post | | -0.026 | -0.09, 0.04 | .430 |  | -0.026 | -0.09, 0.04 | .430 |  | -0.026 | -0.09, 0.04 | .423 |  | -0.026 | -0.09, 0.04 | .423 |  | -0.026 | -0.09, 0.04 | .422 |
| Stimulation x Pre-Dur | | 0.01 | -0.02, 0.04 | .501 |  | 0.01 | -0.02, 0.04 | .501 |  | 0.01 | -0.02, 0.04 | .498 |  | 0.01 | -0.02, 0.04 | .499 |  | 0.01 | -0.02, 0.04 | .496 |
| Stimulation X Pre-Post | | 0.016 | -0.03, 0.06 | .505 |  | 0.016 | -0.03, 0.06 | .505 |  | 0.016 | -0.03, 0.06 | .499 |  | 0.016 | -0.03, 0.06 | .499 |  | 0.016 | -0.03, 0.06 | .498 |
| Site x List | | 0.013 | -0.02, 0.04 | .401 |  | 0.013 | -0.02, 0.04 | .401 |  | 0.013 | -0.02, 0.04 | .400 |  | 0.012 | -0.02, 0.04 | .439 |  | 0.013 | -0.02, 0.04 | .400 |
| Stimulation x List | | -0.005 | -0.03, 0.02 | .711 |  | -0.005 | -0.03, 0.02 | .711 |  | -0.005 | -0.03, 0.02 | .710 |  | -0.005 | -0.03, 0.02 | .710 |  | -0.005 | -0.03, 0.02 | .703 |
| Pre-Dur x List | | 0.107 | 0.05, 0.17 | **<.001** |  | 0.107 | 0.05, 0.17 | **<.001** |  | 0.107 | 0.05, 0.17 | **<.001** |  | 0.109 | 0.05, 0.16 | **<.001** |  | 0.107 | 0.05, 0.17 | **<.001** |
| Pre-Post x List | | 0.046 | 0.02, 0.08 | **.004** |  | 0.046 | 0.02, 0.08 | **.004** |  | 0.046 | 0.02, 0.08 | **.003** |  | 0.046 | 0.02, 0.08 | **.003** |  | 0.046 | 0.02, 0.08 | **.003** |
| Site x Stim x Pre-Dur | | -0.043 | -0.10, 0.01 | .144 |  | -0.043 | -0.10, 0.01 | .144 |  | -0.043 | -0.10, 0.01 | .139 |  | -0.043 | -0.10, 0.01 | .138 |  | -0.043 | -0.10, 0.01 | .138 |
| Site x Stim x Pre-Post | | -0.029 | -0.12, 0.06 | .544 |  | -0.029 | -0.12, 0.06 | .544 |  | -0.029 | -0.12, 0.06 | .539 |  | -0.029 | -0.12, 0.06 | .540 |  | -0.03 | -0.12, 0.06 | .539 |
| Site x Stim x List | | 0.034 | -0.02, 0.09 | .194 |  | 0.034 | -0.02, 0.09 | .194 |  | 0.034 | -0.02, 0.09 | .194 |  | 0.034 | -0.02, 0.09 | .193 |  | 0.034 | -0.02, 0.09 | .194 |
| Site x Pre-Dur x List | | -0.023 | -0.06, 0.02 | .240 |  | -0.023 | -0.06, 0.02 | .240 |  | -0.023 | -0.06, 0.02 | .239 |  | -0.023 | -0.06, 0.02 | .241 |  | -0.023 | -0.06, 0.02 | .240 |
| Site x Pre-Post x List | | -0.005 | -0.04, 0.03 | .804 |  | -0.005 | -0.04, 0.03 | .804 |  | -0.005 | -0.04, 0.03 | .803 |  | -0.005 | -0.04, 0.03 | .806 |  | -0.005 | -0.04, 0.03 | .806 |
| Stim x Pre-Dur x List | | 0.022 | -0.01, 0.06 | .239 |  | 0.022 | -0.01, 0.06 | .239 |  | 0.022 | -0.01, 0.06 | .239 |  | 0.022 | -0.01, 0.06 | .239 |  | 0.022 | -0.01, 0.06 | .234 |
| Stim x Pre-Post x List | | 0.025 | -0.01, 0.06 | .176 |  | 0.025 | -0.01, 0.06 | .176 |  | 0.025 | -0.01, 0.06 | .176 |  | 0.025 | -0.01, 0.06 | .175 |  | 0.025 | -0.01, 0.06 | .173 |
| Site x Stim x Pre-Dur x List | | -0.05 | -0.12, 0.02 | .175 |  | -0.05 | -0.12, 0.02 | .175 |  | -0.05 | -0.12, 0.02 | .174 |  | -0.05 | -0.12, 0.02 | .175 |  | -0.05 | -0.12, 0.02 | .175 |
| Site x Stim x Pre-Post x List | | -0.064 | -0.14, 0.01 | ***.081*** |  | -0.064 | -0.14, 0.01 | ***.081*** |  | -0.064 | -0.14, 0.01 | ***.081*** |  | -0.064 | -0.14, 0.01 | ***.080*** |  | -0.064 | -0.14, 0.01 | ***.081*** |

Table S2 (Continued).

|  |  | Parsimonious LMM |  | Parsimonious + Blinding |  | Parsimonious + Order |  | Parsimonious + Zipf |  | Parsimonious + Baseline RT |
| --- | --- | --- | --- | --- | --- | --- | --- | --- | --- | --- |
| Random Parts | | | | | | | | | | |
| σ^2^ |  | 0.049 |  | 0.049 |  | 0.049 |  | 0.049 |  | 0.049 |
| τ_00, item_ |  | 0.026 |  | 0.026 |  | 0.026 |  | 0.024 |  | 0.026 |
| τ_00, PID_ |  | 0.021 |  | 0.021 |  | 0.022 |  | 0.021 |  | 0.005 |
| ρ_01_ |  | 0.063 |  | 0.063 |  | 0.063 |  | 0.088 |  | 0.062 |
| ICC_item_ |  | 0.272 |  | 0.272 |  | 0.268 |  | 0.257 |  | 0.326 |
| ICC_PID_ |  | 0.222 |  | 0.221 |  | 0.231 |  | 0.226 |  | 0.063 |
| N_item_ |  | 129 |  | 129 |  | 129 |  | 129 |  | 129 |
| N_PID_ |  | 28 |  | 28 |  | 28 |  | 28 |  | 28 |
| Total Observations | | 13775 |  | 13775 |  | 13775 |  | 13775 |  | 13775 |
| R^2^ / Ω_0_^2^ | | .500 / .500 |  | .500 / .500 |  | .500 / .500 |  | .500 / .500 |  | .500 / .500 |
| AIC | | -1563.814 |  | -1561.885 |  | -1564.184 |  | -1566.887 |  | -1666.806 |
