# Supplementary material for: tDCS Facilitation of Picture Naming: Item-Specific, Task General, or Neither?
Source: Front Neurosci. 2018 Aug 10;12:549. doi: 10.3389/fnins.2018.00549 (PMC6095956; doi:10.3389/fnins.2018.00549)
Supplement: Supplementary file 3 [file Table_3.DOCX]

Table S3. *Simple Effects LMMs for IFG Group for B and C Items*

|  | Simple Effects: IFG Group | | |
| --- | --- | --- | --- |
|  | β | 95% CI | *p* |
| (Intercept) | -1.579 | -1.66, -1.50 | <.001 |
| Stimulation (Sham, Active) | -0.034 | -0.10, 0.03 | 0.328 |
| Pre-During | 0.01 | -0.05, 0.08 | 0.754 |
| Pre-Post | -0.079 | -0.13, -0.03 | **0.004** |
| Stimulation*Pre-During | 0.054 | 0.01, 0.10 | **0.011** |
| Stimulation*Pre-Post | 0.061 | 0.01, 0.11 | **0.038** |
|  |  |  |  |
| Random Effects |  |  |  |
| σ^2^ |  |  | 0.051 |
| τ_00, item_ |  |  | 0.031 |
| τ_00, PID_ |  |  | 0.019 |
| ρ_01_ |  |  | -0.933 |
| N_item_ |  |  | 86 |
| N_PID_ |  |  | 14 |
| ICC_item_ |  |  | 0.307 |
| ICC_PID_ |  |  | 0.193 |
| Observations |  |  | 3430 |
| R^2^ / Ω_0_^2^ |  |  | .456 / .455 |
